# Correlation Between Insecure Attachment Style and Symptomatology in Patients With Bipolar Disorder: A Systematic Review

**DOI:** 10.62641/aep.v54i2.2108

**Published:** 2026-04-15

**Authors:** Lorena Suárez, María Cruz-Fortún, María Martín-González, Olaya Tamayo-Morales, Nelson Andrade-González

**Affiliations:** ^1^Faculty of Biomedical and Health Sciences, Alfonso X el Sabio University, 28691 Villanueva de la Cañada, Madrid, Spain; ^2^Mental Health Research Group (MHeRG), Faculty of Biomedical and Health Sciences, Alfonso X el Sabio University, 28691 Villanueva de la Cañada, Madrid, Spain; ^3^Department of Psychology, Faculty of Biomedical and Health Sciences, Universidad Europea de Madrid, 28670 Villaviciosa de Odón, Madrid, Spain; ^4^Primary Care Research Unit of Salamanca (APISAL), Primary Care Management of Salamanca, Regional Health Management of Castilla y León (SACyL), 37008 Salamanca, Spain; ^5^Psychiatry and Mental Health Research Group, Faculty of Medicine and Health Sciences, University of Alcalá, 28801 Alcalá de Henares, Madrid, Spain

**Keywords:** insecure attachment, anxious attachment, avoidant attachment, symptomatology, bipolar disorder

## Abstract

**Background:**

Attachment style describes the bond between a child and their caregivers, and its effect on subsequent relationships. Bipolar disorder (BD) is a mood disorder characterized by fluctuations in mood and energy levels. The aim of this work is to update the evidence on the relationship between two insecure attachment styles (anxious and avoidant) and the symptomatology of patients with BD.

**Methods:**

To achieve the objectives of this systematic review, the Preferred Reporting Items for Systematic Reviews and Meta-Analyses statement guidelines were followed. Searches were conducted from inception until July 24, 2025 in the PsycInfo, PubMed, Scopus, and Web of Science databases. A structured process was carried out for study selection, data extraction, and risk of bias assessment of the included studies.

**Results:**

A total of six articles were included, five of which were cross-sectional and one was a case-control study. These six studies included a total of 466 patients, with a weighted mean age of 41.4 years old. The weighted mean age was calculated according to the sample size of each of the selected articles. The female-to-male ratio was 1.53:1. Patients with insecure attachment experienced depressive, anxiety, and somatization symptoms, as well as greater symptom severity (increased risk of suicidal behavior, greater number of hospitalizations, higher frequency of affective episodes, and greater substance use).

**Conclusions:**

In patients with BD, insecure attachment is associated with greater symptom severity. Future research should investigate the explanatory mechanisms underlying the relationship between insecure attachment and the symptomatology of these patients.

## Introduction

Bipolar disorder (BD) is a chronic and disabling affective disorder whose main characteristic is the presence of fluctuations in mood and energy levels. These fluctuations range from depressive episodes, defined by a depressed mood and notable anhedonia, to manic episodes, characterized by an abnormally elevated or irritable mood [1].

Regarding its diagnosis, both the Diagnostic and Statistical Manual of Mental Disorders (DSM) [2] and the International Classification of Diseases (ICD) [1] distinguish three main categories: (1) BD type I, defined by the presence of at least one manic episode, without the requirement of major depressive episodes; (2) BD type II, defined by the occurrence of at least one major depressive episode along with a hypomanic episode, without a history of manic episodes; and (3) cyclothymic disorder, characterized by chronic mood fluctuations that do not meet the diagnostic criteria in terms of duration or intensity to be classified as manic or major depressive episodes.

Likewise, both the World Health Organization [1] and the American Psychiatric Association [2] concur that BD is a psychopathological mood disorder whose course is associated with a high likelihood of relapse, marked impairment in overall functioning, high comorbidity with other psychiatric disorders, and according to Pompili *et al*. [3] a risk of suicidal behavior up to 20–30 times greater than that of the general population.

The World Health Organization [1] emphasizes that BD constitutes a considerable burden on public health, being one of the leading causes of disability with regards to mental health. In 2019, it was estimated that approximately 40 million people, equivalent to 0.53% of the globally adult population, were suffering from this disorder [1].

According to Bowlby [4, 5] and Ainsworth [6, 7], attachment describes the effect that early experiences and the relationship with the primary attachment figure, such as parents or caregivers, have on development. Following these authors, there are three main styles of attachment in childhood: secure, anxious, and avoidant [4, 5, 6, 7], although the disorganized style was identified more recently [8].

The secure attachment style develops when the caregiver is consistent, available, and responsive to the child’s needs [4, 5]. Conversely, the anxious attachment style (also referred to as ambivalent, or anxious-ambivalent) emerges from the need to obtain the attention of an inconsistent caregiver [4, 9]. In turn, the avoidant attachment style develops from the need to inhibit emotional expression, given its limited effectiveness in eliciting a response from caregivers [4, 9]. Finally, the disorganized attachment style presents characteristics of both the anxious and avoidant styles. It develops as a result of disruptive experiences—such as neglect or physical and sexual abuse in childhood—where the caregiver is simultaneously a source of comfort and fear for the child [8, 10].

According to Bowlby’s attachment theory [5], the experiences maintained by children with their caregiving figures are progressively internalized. This internalization is crucial, as it establishes a prototype that structures early attachment relationships, serving as a relational template for subsequent social interactions that the individual will establish outside the family context. Bowlby [11] identified two fundamental dimensions of these internal representations, known as Internal Working Models: self-image (the image the child holds of themselves) and the representation of the other (the image the child holds of other people).

Building upon this conceptualization of the Internal Working Models, Bartholomew and Horowitz [12] and Griffin and Bartholomew [13] developed a classification system for adult attachment. This system is based on the logical derivation of four categories resulting from the bidimensional combination of two axes: the level of self-image (positive versus negative) and the level of the image of others (positive versus negative). This resultant matrix yields the four main adult attachment styles: secure, preoccupied, dismissing, and fearful.

In adulthood, individuals with a secure attachment style hold a positive view of both themselves and others, which allows them to feel comfortable with intimacy without compromising their personal autonomy and to maintain an internalized and stable sense of self-esteem [12, 14]. However, preoccupied attachment (which corresponds to anxious attachment style), is defined by a negative self-view and a positive model of others, which is associated with greater dependency and separation anxiety, excessive involvement in close relationships, and insufficient emotional regulation [12, 14, 15, 16]. Furthermore, these individuals exhibit an amplified appraisal of potential threats, frequently attributing such events to uncontrollable factors or global personal deficits [17]. In contrast, dismissing attachment (which corresponds to avoidant attachment style), is characterized by a positive view of the self and a negative view of others [12, 18, 19]. People with this style find it difficult to trust others enough to genuinely bond with them and are reluctant to feel and express emotions that lead them to connect with others [20]. When experiencing distress, they emphasize self-reliance and frequently ignore or suppress negative affect [21]. Finally, fearful attachment is characterized by a negative self-model and a negative model of others, along with the inability to develop coherent strategies for emotional regulation [10, 12, 18, 22]. Individuals with fearful attachment style oscillate between seeking proximity and rejecting intimacy, a conflict that frequently undermines relational stability [22].

### Objectives

Although the influence of an insecure attachment style in the general population and in patients with other disorders is well documented, its specific impact on the complex and fluctuating symptomatology of BD has not yet been adequately synthesized. Therefore, the primary aim of this review is to provide updated evidence on the association between two insecure attachment styles (anxious and avoidant), and the symptomatology of patients with BD. Accordingly, the research question guiding this review is: Is there a relationship between insecure attachment style and the symptoms exhibited by patients with BD? The specific objectives are as follows: (1) to examine the association of anxious and avoidant attachment styles on the severity of symptoms in patients with BD, specifically suicidal behavior, levels of depression, anxiety, and somatization, number of hospitalizations, frequency of affective episodes, and substance use; and (2) to determine whether there are differences in the symptoms of patients with BD based on either type of insecure attachment style (anxious or avoidant). Consequently, the hypotheses are as follows: (1) the insecure attachment style of patients with BD will be related to heightened severity of clinical symptoms, characterized by elevated levels of depression, anxiety and somatization, as well as an increased risk of suicidal behavior, greater number of hospitalizations, and higher substance use; and (2) there will be differences in the symptoms of patients with BD depending on their insecure attachment style. Individuals with an anxious attachment style will exhibit higher levels of depressive symptomatology and suicidal behaviors. Conversely, individuals with an avoidant attachment style will be predisposed to eschew help-seeking behaviors and to exhibit elevated levels of somatization.

## Methods

To achieve the objectives of this systematic review, the recommendations of the Preferred Reporting Items for Systematic Reviews and Meta-Analyses (PRISMA) statement [23] were followed. A search was conducted for systematic reviews and meta-analyses on the topic of this systematic review, and it was found that no such work had been published to date. In addition, the 27-item PRISMA checklist (see **Supplementary Table 1**) and structured abstract checklist (see **Supplementary Table 2**) were applied.

### Literature Search

In order to identify relevant literature, searches were conducted in the PsycInfo, Web of Science (WoS), Scopus, and PubMed databases. The search strategy employed in each of these databases followed the Population, Exposure, and Outcomes (PEO) framework, that is: Population (patients with BD type I, BD type II, or cyclothymia); Exposure (patients with a specific attachment style); and Outcomes (clinical symptomatology). Prior to conducting the database searches, the following Medical Subject Headings (MeSH) were used: *bipolar disorder* and *cyclothymic disorder*. Keywords were additionally combined through the use of Boolean operators. An example of a search strategy was as follows: *attachment* OR “*attachment style*” OR “*attachment theory*” AND *patient* OR *client* AND “*bipolar disorder*” OR “*bipolar depression*” OR “*manic depression*” OR “*bipolar affective disorder*” (see **Supplementary Table 3**). Regarding filters, the language filter was restricted to English. The database searches covered the period from inception until July 24, 2025. In addition, a manual search was conducted in Google Scholar, and the reference lists of the studies ultimately included were reviewed.

### Inclusion and Exclusion Criteria

To verify the inclusion and exclusion criteria of the articles, a checklist was developed (see **Supplementary Table 4**). The inclusion criteria were as follows: (1) participation of patients over 18 years of age with a diagnosis of BD type I, BD type II, or cyclothymic disorder; (2) patients with a specific attachment style; (3) use of attachment style measures completed either by the patient or by the investigator; (4) experimental, quasi-experimental, or observational studies; (5) publications in English; and (6) studies published from inception until July 24, 2025. The exclusion criteria were: (1) patients with unipolar depression and/or psychotic disorders; (2) articles that included patients with BD and other diagnoses, but did not separate the results based on those diagnoses; (3) systematic reviews and meta-analyses, qualitative studies, single case reports, case series, letters to the editor, opinion papers or commentaries, brief communications, and book chapters; (4) conference abstracts; and (5) studies that did not use a validated attachment measure.

### Study Selection Process

**Fig. 1.**
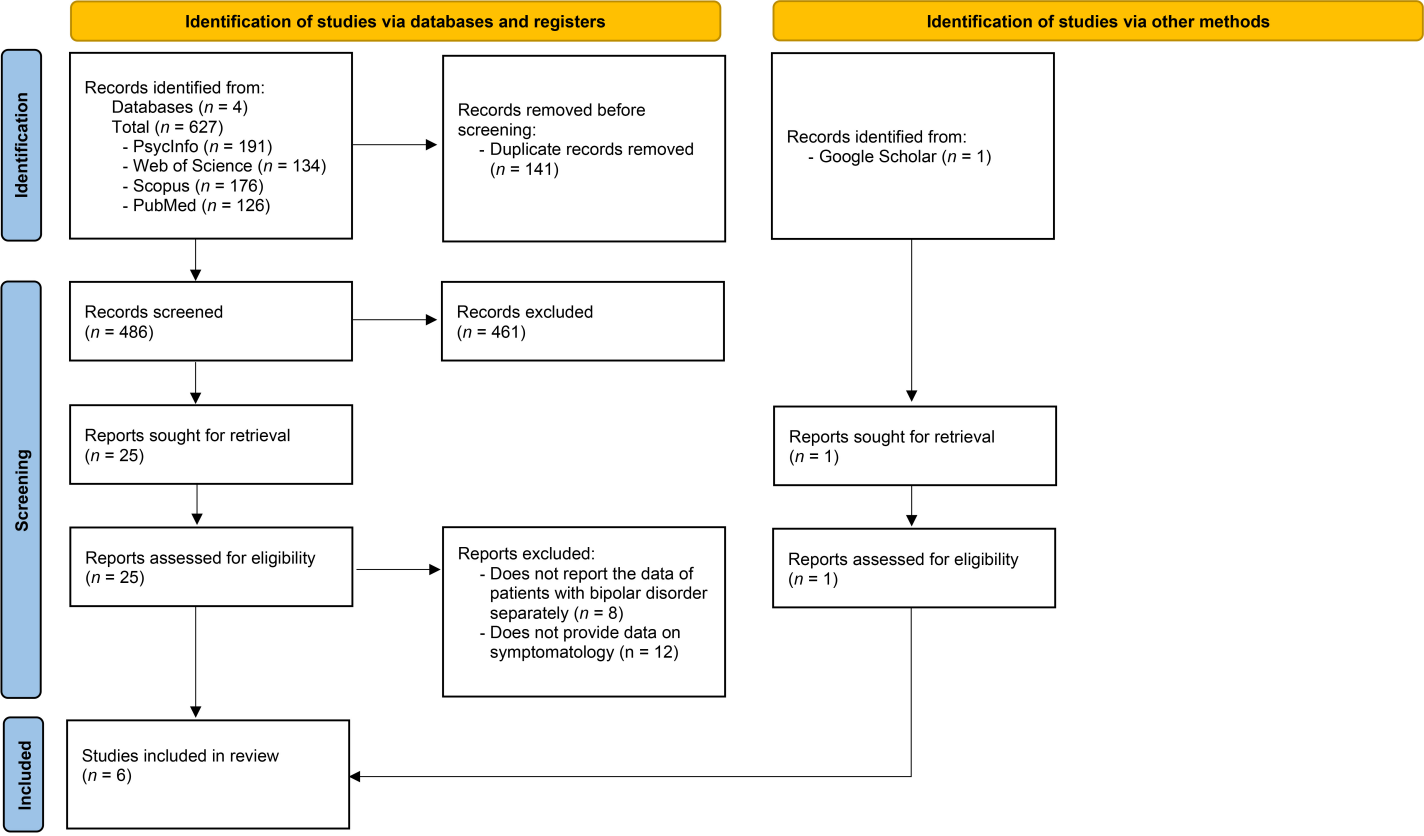
**The Preferred Reporting Items for Systematic Reviews and Meta-analyses’ diagram that illustrates the process of selecting the articles for the systematic review**.

The selection process was divided into four stages and carried out by two independent reviewers (L.S. and N.A-G.). These two reviewers separately analyzed all the articles. Disagreements in any stage of the selection process were resolved by an expert reviewer (N.A-G.). In the identification phase (conducted on July 24, 2025), the 627 results retrieved from the four databases were consolidated and duplicates were removed using Zotero bibliographic management software [24]. During the screening phase, the titles and abstracts of 486 articles that potentially met the previously defined inclusion criteria were examined. The 461 studies that did not address the topic of this systematic review in their title or abstract were excluded. Articles with unclear content were advanced to the next stage. In the eligibility phase, the full texts of 25 articles preselected in the screening phase were assessed. At this stage, it was verified whether these studies met the inclusion criteria of the present review. In addition, further studies were sought in the reference lists of the selected articles and through Google Scholar. Finally, in the inclusion phase, 6 articles were selected to comprise this systematic review (see Fig. 1).

### Data Extraction Process

To analyze and synthesize the information, a critical reading of the six selected articles was undertaken. The following data were extracted from these studies: (1) authors and year of publication; (2) sample size and mean age of participants; (3) instrument used to assess attachment style; (4) instrument used to assess symptomatology; (5) data used to determine study design; (6) data used to evaluate study risk of bias; and (7) main findings.

### Risk of Bias Assessment

The risk of bias of the included studies was assessed using the Joanna Briggs Institute (JBI) Critical Appraisal Checklists [25]. The Joanna Briggs Institute aims to support evidence–based practice across different healthcare fields [26]. Risk of bias was appraised by two independent reviewers (L.S. and N.A-G.). Since this systematic review includes five cross–sectional studies and one case–control study, two checklists were used. Both instruments consist of questions to be answered with “Yes”, “No”, “Unclear”, or “Not applicable”. For cross–sectional studies, the Joanna Briggs Institute Critical Appraisal Checklist for Analytical Cross-Sectional Studies was used [25], which comprises 8 items (see **Supplementary Table 5**). For case–control studies, the 10-item Joanna Briggs Institute Critical Appraisal Checklist for Case–Control Studies was applied [25] (see **Supplementary Table 6**).

### Method of Analysis and Data Synthesis

A meta-analysis could not be performed due to the lack of a common effect size. The studies reported heterogeneous and non–comparable results: Pearson (*n* = 3) and Spearman (*n* = 1) correlations on different constructs, as well as categorical statistics (χ^2^). This heterogeneity prevents the standardization of effects and the calculation of indicators such as *I*^2^, so a narrative synthesis was chosen. The tables and figures were created using version 16.103.4 of Microsoft Word by Microsoft Corporation [27].

## Results

Six original studies that met the inclusion criteria were selected [28, 29, 30, 31, 32, 33]. The selected studies included a total of 466 patients with a mean age of 41.4 years old (*k* = 6). The weighted mean age was calculated according to the sample size of each of the selected articles. The female-to-male ratio was 1.53:1. Fig. 1 shows the selection process for these studies.

### Characteristics of the Included Studies

Table 1 [28, 29, 30, 31, 32, 33] presents the key characteristics of the six studies included in this systematic review. The following details are provided: (1) authors and year of publication; (2) country where the study was conducted; (3) study title; (4) sample characteristics; (5) diagnosis criteria; (6) evaluated variables; (7) study design; and (8) main findings.

**Table 1.** **Characteristics and main results of the studies selected in the present systematic review**.

Authors, year	Country	Title	Sample characteristics	Diagnosis criteria	Evaluated variables	Study design	Main results
Citak and Erten (2021) [28]	Turkey	Impact of Childhood Trauma and Attachment on Resilience in Remitted Patients with Bipolar Disorder	*N* = 110	DSM-5	- Attachment style: Experiences in Close Relationships-Revised (ECR-R)	Cross-sectional	Correlations
			- Female: *n *= 53				1. Anxious attachment and HAM-D (*rho *= 0.271, *p* < 0.01)
			- Male: *n* = 57				
			- BD-I: *n* = 100		- Severity of depression: The Hamilton Depression Rating Scale (HAM-D)		
			- BD-II: *n* = 10				
			*M*_𝑎𝑔𝑒_ = 37.21				
Gilbert *et al*. (2007) [29]	United Kingdom	Social Rank and Attachment in People with a Bipolar Disorder	*N* = 40	ICD-10	- Attachment style: Adult Attachment Scale (AAS)	Cross-sectional	Correlations:
			- Female: *n *= 21				1. Close Subscale (AAS-C)
			- Male: *n* = 19		- Severity of depression: The Beck Depression Inventory (BDI)		a. BDI (*r* = –0.40, *p * < 0.05)
			*M*_𝑎𝑔𝑒_ = 46.8				b. ISS-ACT (*r* = –0.04)
					- Severity of manic and depressive symptoms: Internal State Scale (ISS)		c. ISS-PC (*r* = –0.22)
							d. ISS-WB (*r* = 0.38, *p * < 0.05)
							e. ISS-DI (*r =* –0.29)
							2. Anxious Subscale (AAS-A)
							a. BDI (*r* = 0.27)
							b. ISS-ACT (*r* = 0.29)
							c. ISS-PC (*r* = 0.22)
							d. ISS-WB (*r* = –0.11)
							e. ISS-DI (*r* = 0.32, *p * < 0.05)
							3. Depend Subscale (AAS-D)
							a. BDI (*r* = –0.03)
							b. ISS-ACT (*r* = –0.05)
							c. ISS-PC (*r* = –0.09)
							d. ISS-WB (*r* = 0.10)
							e. ISS-DI (*r* = 0.02)
Kökçü and Kesebir (2010) [30]	Turkey	The Relationship between Attachment Style, and Temperament, Personality and Bipolar Symptoms: A Controlled Study on Bipolar Patients and Their Children	*N *= 128	DSM-III-R	- Attachment style: Adult Attachment Scale (AAS)	Case-control	Insecure attachment and clinical characteristics
			Adults with BD: *n* = 44	DSM-IV			
			- Female: *n* = 28		- Specific symptom domains: Structured Clinical Interview for DSM-Axis I Disorders (SCID-I), Structured Clinical Interview for DSM-Axis II Disorders (SCID-II), Diagnostic and Monitoring Form for Mood Disorders (SCIP-TURK)		1. Premenstrual syndrome (*p* = 0.008, χ^2^ = 14.825, *SD* = 1)
			- Male: *n *= 16				
			- BD-I: *n* = 36				2. Severe manic/depressive episodes (*p* = 0.028, χ^2^ = 9.456, *SD* = 2)
			- BD-II: *n* = 8				
			*M*_𝑎𝑔𝑒_ = 40.7				3. Postpartum onset (*p* = 0.052, χ^2^ = 1532, *SD* = 1)
			Control group: *n* = 84				
			*M*_𝑎𝑔𝑒_ = 32.6				4. Seasonality (*p* = 0.029, χ^2^ = 7562, *SD* = 1)
							5. Depression-mania-remission pattern (*p* = 0.039, χ^2^ = 7685, *SD* = 7)
							6. Sudden onset (*p* = 0.039, χ^2^ = 7430, *SD* = 1)
							7. Number of hospitalizations (*p* = 0.039, *t* = 2.7, *SD* = 8)
							8. Alcohol use (*p* = 0.034, χ^2^ = 8125, *SD *= 1)
							9. Drug use (*p* = 0.051, χ^2^ = 1248, *SD *= 1)
							10. Poor social functioning (*p* = 0.007, χ^2^ = 14.520, *SD* = 3)
Morán-Kneer *et al*. (2022) [31]	Chile	Childhood Trauma and Social Cognition in Participants with Bipolar Disorder: The Moderating Role of Attachment	*N* = 76	DSM-IV-TR	- Attachment style: Experiences in Close Relationships (ECR)	Cross-sectional	Greater number of hospitalizations in the insecure attachment style compared to the secure attachment style (*U* = 382, *p* = 0.014)
			- Female: *n* = 53				
			- Male: *n *= 23		- Number of hospitalizations		
			- BD-I: *n *= 76				
			*M*_𝑎𝑔𝑒_ = 46.3				
Şen and Yildizhan (2020) [32]	Turkey	Relationship of Intolerance of Uncertainty and Attachment Styles with the Clinical Features of Bipolar Disorder in Remission	*N *= 150	DSM-5	- Attachment style: The Inventory of Close Relationship Experiences 2 (ICRE-2)	Cross-sectional	Correlations
			- Female: *n* = 94				1. Anxious attachment
			- Male: *n *= 56				a. IUS-12 prospective anxiety (*r *= 0.398, *p* < 0.01)
			*M*_𝑎𝑔𝑒_ = 37.33		- Intolerance of Uncertainty: The Intolerance of Uncertainty Scale 12 (IUS-12)		
							b. IUS-12 inhibitory anxiety (*r* = 0.371, *p* < 0.01)
					- Suicidal Behavior: The Suicidal Behavior Questionnaire (SBQ)		c. SBQ (*r* = 0.264, *p * < 0.001)
							2. Avoidant attachment
							a. IUS-12 prospective anxiety (*r *= –0.061)
							b. IUS-12 inhibitory anxiety (*r* = 0.084)
							c. SBQ (*r* = 0.148)
							Post hoc analysis
							1. Patients with 2 or more suicide attempts (*X* = 4.32) scored significantly higher on avoidant attachment compared to patients with no ideation (*X* = 3.01), ideation without attempts (*X* = 3.01), and one attempt (*X* = 3.17)
Wagner-Skacel *et al*. (2020) [33]	Austria	Personality Structure and Attachment in Bipolar Disorder	*N* = 46	DSM-IV	- Attachment style: Experiences in Close Relationships-Revised (ECR-R)	Cross-sectional	Correlations
			- Female: *n *= 21				1. Anxious attachment
			- Male: *n* = 25				a. BSI18-Depression (*r* = 0.47, *p * < 0.01)
			- BD-I: *n *= 23		- Psychiatric Symptoms and Psychological Distress: Brief Symptom Inventory (BSI-18)		b. BSI18-Anxiety (*r* = 0.27)
			- BD-II: *n* = 23				c. BSI18-Somatization (*r *= 0.25)
			*M*_𝑎𝑔𝑒_ = 47.4				d. BSI18-Total (*r* = 0.40, *p * < 0.01)
							2. Avoidant attachment
							a. BSI18-Depression (*r* = 0.42, *p * < 0.01)
							b. BSI18-Anxiety (*r* = 0.35, *p * < 0.05)
							c. BSI18-Somatization (*r* = 0.43, *p * < 0.01)
							d. BSI18-Total (*r* = 0.47, *p * < 0.01)

*Note*: BD-I, Bipolar Disorder Type 1; BD-II, Bipolar Disorder Type 2; *M*_𝑎𝑔𝑒_, Mean age; DSM, Statistical Manual of Mental Disorders; ICD, International Classification of Diseases; AAS-C, Adult Attachment Scale-Close; ISS-ACT, Internal State Scale-Activation; ISS-PC, Internal State Scale-Personal Conflict; ISS-WB, Internal State Scale-Well-Being; ISS-DI, Internal State Scale-Depression Index; AAS-A, Adult Attachment Scale-Anxious; AAS-D, Adult Attachment Scale-Depend; BD, Bipolar Disorder; *SD*, Standard Deviation.

Attachment style was assessed with the following validated instruments: two studies employed the Experiences in Close Relationships–Revised (ECR–R) [34]; two studies used the Adult Attachment Scale (AAS) [35]; one study used the Experience in Close Relationship (ECR) [36]; and one study used the Inventory of Close Relationship Experiences–2 (ICRE–2) [37].

### Risk of Bias

The risk of bias assessment of the included cross-sectional and case-control studies is presented below (see Table 2 [28, 29, 31, 32, 33] and Table 3 [30]). Regarding the results of the risk of bias analysis, all studies were categorized as low risk of bias.

**Table 2.** **Risk of bias assessment of cross–sectional studies using the JBI Critical Appraisal Checklist**.*

Study	1	2	3	4	5	6	7	8
Citak and Erten (2021) [28]								
Gilbert *et al*. (2007) [29]								
Moran-Kneer *et al*. (2022) [31]								
Şen and Yildizhan (2020) [32]								
Wagner-Skacel *et al*. (2020) [33]								

*Note.* * = The full description of the items can be found in **Supplementary Table 5**. ■ Low risk of bias ■ Unclear ■ High risk of bias

**Table 3.** **Risk of bias assessment of case–control studies using the JBI Critical Appraisal Checklist**.*

Study	1	2	3	4	5	6	7	8	9	10
Kökçü & Kesebir (2010) [30]										

*Note.* * = The full description of the items can be found in **Supplementary Table 6**. ■ Low risk of bias ■ Unclear ■ High risk of bias

### Synthesis of Results

Regarding the symptomatology of patients with BD, an increased symptomatology has been consistently associated with an insecure attachment (as a global construct) in two separate reports [30, 31]. More specifically, four studies linked the characteristic symptoms of BD to an anxious attachment style [28, 29, 32, 33], while two studies demonstrated this association with an avoidant attachment style [32, 33].

Focusing on specific symptom domains, Citak and Erten [28], Gilbert *et al.* [29], and Wagner-Skacel *et al*. [33] all observed a statistically significant relationship between an anxious attachment style and the presence of depressive symptoms. Furthermore, Wagner-Skacel *et al*. [33] reported that an avoidant attachment style was associated with depressive, anxiety, and somatization symptoms. Kökçü and Kesebir [30] further documented that insecure attachment (globally considered) was related to a greater frequency of severe affective episodes.

In the domain of suicidal behavior, Şen and Yildizhan [32] found that anxious attachment was significantly correlated with both intolerance of uncertainty and suicidal behavior. A post hoc analysis conducted by the same authors revealed that patients who reported two or more suicide attempts scored significantly higher on avoidant attachment compared to those with no history of suicidal ideation or attempts.

Regarding other indices of illness burden, the two studies examining overall insecure attachment [30, 31] found that BD patients experienced a higher frequency of hospitalizations. Notably, Kökçü and Kesebir [30] also identified that this insecure attachment style was associated with greater substance use, a higher prevalence of premenstrual syndrome, and more pronounced impairment in social functioning.

## Discussion

In line with the primary objective of this review, all six included studies evidenced an association between patients’ insecure attachment style and the symptomatology of BD [28, 29, 30, 31, 32, 33].

In relation to the anxious attachment style, the works of Citak and Erten [28] and Wagner-Skacel *et al*. [33] point out that fear of rejection and separation anxiety have been associated with the development of depressive symptoms in patients with BD. This vulnerability stems from low self-confidence regarding one’s worth as a person deserving of affection and from the persistent hyperactivation of the attachment system [28]. Additionally, the findings of Gilbert *et al*. [29] indicate a significant correlation between an anxious attachment style and the manifestation of depressive symptoms. This study emphasize that mood regulation in this population is strongly influenced by the perception of social rank and associated behaviors. It is proposed that the relationship between attachment and social hierarchy is complex: individuals who struggle to establish secure attachment may adopt competitive strategies to gain recognition and control over resources within their social environment, thereby seeking to compensate for relational insecurity. In addition, the study by Şen and Yildizhan [32] observes for the first time a relationship between intolerance to uncertainty and anxious attachment, which could be explained by these patients’ tendency to perceive uncertainty as a threat of abandonment. Lastly, the study by Morán-Kneer *et al*. [31] reported consistent findings, identifying anxious attachment as a moderating variable in the relationship between early traumatic experiences and social cognition in individuals with BD.

With regard to patients with an avoidant attachment style, Şen and Yildizhan [32] suggested that they may have low motivation to seek help, which could limit their access to and the effectiveness of interventions in the event of suicidal behaviors or attempts. Furthermore, Wagner-Skacel* et al*. [33] noted that a lack of self-disclosure and a marked self-absorption may be associated with greater psychological distress, manifested as somatization, depression, or anxiety.

In general, BD patients with an insecure attachment style tend to show difficulties in interpersonal relationships, higher levels of stress, and more severe symptomatology, which increases their vulnerability and reduces their coping strategies in crisis situations [33]. In line with this, Kökçü and Kesebir [30] emphasized the importance of establishing a robust working alliance with these patients, considering it a key element for addressing these difficulties and facilitating the recovery process.

Regarding the severity of symptomatology and the course of the disorder, several studies have indicated that insecure attachment is associated with a higher number of hospitalizations [30, 31], increased substance use, greater impairment in social functioning [30], and a higher number of suicide attempts [32]. In this context, other studies support the hypothesis that insecure attachment styles may act as moderating variables in the impact of early trauma or adversity on the course of the disorder, thereby exacerbating its severity [28, 31]. Likewise, one study point to an indirect relationship between insecure attachment and the symptomatology of BD. Both insecure styles (anxious and avoidant) acted as partial mediators of the negative impact that childhood trauma exerts on resilience in BD patients [28].

The attachment style has also been examined within the spectrum of psychotic disorders. In the meta-analysis by Carr *et al*. [38], insecure attachment was found to be significantly more prevalent among individuals with psychosis (76%) compared to healthy controls (38%). Similarly, in another meta-analysis on recovery from psychosis, van Bussel *et al*. [39], observed that anxious and avoidant attachment styles were associated with poorer outcomes in personal, social, and symptomatic recovery (positive and general symptoms). In addition, the systematic review by Korver-Nieberg *et al*. [40] reported that insecure attachment was associated with psychotic phenomenology and with greater vulnerability to developing maladaptive coping strategies during recovery from psychosis. Likewise, the systematic review by Gumley *et al*. [41], conducted with patients with severe mental illness, identified an association between insecure attachment and higher levels of positive, negative, and affective symptoms. Finally, the review by Berry *et al*. [42] on adult attachment style in psychosis, found that insecure attachment was related to poorer interpersonal relationships and less integrative recovery styles. In sum, in patients with psychotic disorders, an insecure attachment style is associated with poorer recovery, worse coping strategies, and poorer social functioning. Therefore, these results suggest that the findings regarding the association of insecure attachment in BD are similar to those observed in psychotic disorders.

Regarding implications for clinical practice, it is suggested that: (1) attachment style should be assessed in patients with BD, as insecure attachment (anxious or avoidant) is linked to markers of severity and poor longitudinal prognosis such as a greater number of suicidal behaviors, greater anxious, depressive, and somatic symptomatology, a higher number of hospitalizations, increased frequency of affective episodes, and greater substance use; and (2) this correlational evidence suggests that attachment is a key psychopathological mechanism in BD. Consequently, psychotherapeutic interventions aimed at modulating internal attachment representations and stabilizing affect regulation represent a priority therapeutic approach.

This systematic review has several limitations that should be taken into account when interpreting the findings: (1) the number of studies that have examined the topic of our systematic review to date is limited; (2) differences exist in the patient samples of the included studies (e.g., varying sample sizes and ages); (3) in three studies it was not specified whether patients were in a euthymic phase or experiencing a manic or depressive episode; (4) in all included studies, attachment style measures were self-reported by patients, which may introduce social desirability bias; and (5) all included studies were published in English, which may represent a publication bias.

## Conclusions

The conclusions of the present systematic review are as follows: (1) in patients with BD, a relationship is observed between insecure attachment and symptomatology; (2) in patients with BD, insecure attachment styles (anxious and avoidant) are associated with depressive, anxiety, and somatization symptoms, and also, with greater symptom severity (increased risk of suicidal behavior, greater number of hospitalizations, higher frequency of affective episodes, and greater substance use); and (3) differences in symptomatology are observed in patients with BD depending on the insecure attachment style (anxious or avoidant).

Accordingly, the following future research directions are proposed: (1) conducting studies aimed at elucidating the mechanisms underlying the relationship between insecure attachment and symptomatology in patients with BD; (2) considering the role of age and the family and social environment in the insecure attachment–symptomatology relationship in BD; (3) given the gender ratio observed in this study, future research should examine how gender affects the association between insecure attachment and clinical symptoms in patients with BD; (4) undertaking studies with larger sample sizes to improve the statistical power of findings; and (5) reaching consensus regarding the terminology and core characteristics of attachment styles, in order to avoid ambiguities and enhance communication, collaboration, and comparability of results.

## Availability of Data and Materials

The data supporting the findings of this study are available from the corresponding author upon reasonable request.
