# Supplementary material for: Correlation Between Insecure Attachment Style and Symptomatology in Patients With Bipolar Disorder: A Systematic Review
Source: Actas Esp Psiquiatr. 2026 Apr 15;54(2):516–27. doi: 10.62641/aep.v54i2.2108 (PMC13180678; doi:10.62641/aep.v54i2.2108)
Supplement: Supplementary file 1 [file ActEsp-54-2-516-527-s1.zip › Supplementary Table 3.docx]

**Supplementary** **Table S3. Database search strategies.**

| Databases | Search strategy | Results | Selected |
| --- | --- | --- | --- |
| PsycInfo | XB ((("bipolar disorder" OR "bipolar I" OR "bipolar II" OR "manic depression" OR "bipolar affective disorder" OR "bipolar depression" OR mania OR cyclothymi* OR hypomani* OR manic-depressi*))) AND ("attachment theory" OR attachment OR "attachment style*" OR "attachment pattern*" OR "attachment relationship*"))) | 191 | 3 |
| Web of Science | (ALL=("attachment" OR "attachment theory" OR "attachment style*" OR "attachment relationship*")) AND TI=("bipolar" OR "bipolar disorder" OR "bipolar I" OR "bipolar II" OR "bipolar type 1" OR "bipolar type 2" OR "manic-depressi*" OR "cyclothym*" OR "hypomani*") | 134 | 1 |
| Scopus | ( ( TITLE-ABS-KEY ( bipolar OR mani* OR cyclothymi* OR manic-depressi* OR hypomani* OR bipolar AND disorder ) AND TITLE-ABS-KEY ( object AND attachment OR attachment AND theory OR attachment AND style OR attachment ) ) AND ( LIMIT-TO ( SUBJAREA , "PSYC" ) OR LIMIT-TO ( SUBJAREA , "MEDI" ) ) AND ( LIMIT-TO ( LANGUAGE , "English" ) ) | 176 | 0 |
| PubMed | ((bipolar disorder or bipolar depression[MeSH Terms]) AND (cyclothym* or depressi* or manic-depressi* or hypomani* or bipolar type 1 or bipolar type 2)) AND (attachment or attachment style or attachment theory or attachment relationship) | 126 | 1 |
| Total |  | 486* | 6** |

*Note: **After removing duplicates ** One additional study identified through Google Scholar was also included in the selection.
