# Supplementary material for: Correlation Between Insecure Attachment Style and Symptomatology in Patients With Bipolar Disorder: A Systematic Review
Source: Actas Esp Psiquiatr. 2026 Apr 15;54(2):516–27. doi: 10.62641/aep.v54i2.2108 (PMC13180678; doi:10.62641/aep.v54i2.2108)
Supplement: Supplementary file 1 [file ActEsp-54-2-516-527-s1.zip › Supplementary Table 4.docx]

**Supplementary Table S4 . Inclusion and exclusion criteria checklist systematic review.**

| Checklist item | | Inclusion criteria | Exclusion criteria |
| --- | --- | --- | --- |
| **Population** | |  |  |
|  | | - Adults (aged 18 or older) | - Individuals under 18 years old |
|  | | - Adults diagnosed with BD type I, BD type II, or cyclothymic disorder | - Without a diagnosis of BD - Patients with unipolar depression and/or psychotic disorders |
|  |  | | - Articles that included patients with BD and other diagnoses, but did not separate the results based on those diagnoses |
| **Exposure** |  | |  |
|  | - Patients with a specific attachment style | | - Other types of exposure |
| **Outcome measures** |  | |  |
|  | - Measures of the patient’s attachment style, completed either by the patient or by the investigator | | - Studies that did not use a validated attachment measure |
| **Study design** |  | |  |
|  | - Experimental studies - Quasi-experimental studies - Observational studies | | - Qualitative studies - Single case reports - Case series |
|  |  | | - Systematic reviews and meta-analyses - Letters to the editor - Book chapters - Opinions papers or commentaries - Brief communications |
| **Language** |  | |  |
|  | - English | | - Other languages |
| **Publication date** | - Prior to July 24, 2025 | | - After July 24, 2025 |

*Note* = BD, Bipolar Disorder.
