# Supplementary material for: Correlation Between Insecure Attachment Style and Symptomatology in Patients With Bipolar Disorder: A Systematic Review
Source: Actas Esp Psiquiatr. 2026 Apr 15;54(2):516–27. doi: 10.62641/aep.v54i2.2108 (PMC13180678; doi:10.62641/aep.v54i2.2108)
Supplement: Supplementary file 1 [file ActEsp-54-2-516-527-s1.zip › Supplementary Table 5.docx]

**Supplementary Table S5.** **Risk of bias assessment in cross**–**sectional studies.**

| Items |
| --- |
| 1. Were the criteria for inclusion in the sample clearly defined? |
| 1. Were the study subjects and the setting described in detail? |
| 1. Was the exposure measured in a valid and reliable way? |
| 1. Were objective, standard criteria used for measurement of the condition? |
| 1. Were confounding factors identified? |
| 1. Were strategies to deal with confounding factors stated? |
| 1. Were the outcomes measured in a valid and reliable way? |
| 1. Was appropriate statistical analysis used? |
