# Supplementary material for: Correlation Between Insecure Attachment Style and Symptomatology in Patients With Bipolar Disorder: A Systematic Review
Source: Actas Esp Psiquiatr. 2026 Apr 15;54(2):516–27. doi: 10.62641/aep.v54i2.2108 (PMC13180678; doi:10.62641/aep.v54i2.2108)
Supplement: Supplementary file 1 [file ActEsp-54-2-516-527-s1.zip › Supplementary Table 6.docx]

**Supplementary Table S6.** **Risk of bias assessment in case**–**control studies.**

| Items |
| --- |
| 1. Were the groups comparable other than the presence of disease in cases or the absence of disease in controls? |
| 1. Were cases and controls matched appropriately? |
| 1. Were the same criteria used for identification of cases and controls? |
| 1. Was exposure measured in a standard, valid and reliable way? |
| 1. Was exposure measured in the same way for cases and controls? |
| 1. Were confounding factors identified? |
| 1. Were strategies to deal with confounding factors stated? |
| 1. Were outcomes assessed in a standard, valid and reliable way for cases and controls? |
| 1. Was the exposure period of interest long enough to be meaningful? |
| 1. Was appropriate statistical analysis used? |
